# A characterization study of the anterior segment morphology of patients with and without long-term silicone oil following vitrectomy

**DOI:** 10.1007/s10792-025-03407-7

**Published:** 2025-01-24

**Authors:** Gurfarmaan Singh, Yong Min Lee, Michelle Sun, Dinesh Selva, WengOnn Chan

**Affiliations:** 1 The University of Adelaide, North Terrace, Adelaide, SA 5000 Australia; 2 Department of Ophthalmology, Royal Adelaide Hospital, Port Road, Adelaide, SA 5000 Australia

**Keywords:** Vitrectomy, Glaucoma, Anterior segment, Pathogenesis

## Abstract

**Purpose:**

To characterize the anterior segment (AS) morphology of patients with long-term silicone oil (SiO) in situ (> 12 months) following pars plana vitrectomy (PPV).

**Methods:**

This prospective, comparative characterization study was conducted between January 2022 and July 2023. Patients were included and sorted based on if they had undergone PPV without long-term SiO or had SiO in situ for at least 12 months at the time of review and image collection. The Zeiss Cirrus HD-OCT (Carl Zeiss, Dublin, California, USA) was employed to image the ASs of study patients. Software within the OCT was used to collect the central corneal thickness (CCT), anterior chamber diameter (ACd), anterior chamber depth (ACD), chamber area, temporal irideocorneal angle and nasal irideocorneal angle.

**Results:**

A total of 78 eyes from 42 total patients were included, their mean age was 63.74 ± 19.13 years and 64.29% (27/42) of participants were males. Patients who underwent PPV with and without long-term SiO were found to have no significant difference in CCT, ACd, ACD, chamber area and irideocorneal angles compared to their fellow unoperated eyes. The multivariate analysis of variance of the pooled data noted a statistically significant difference in the ACd of PPV with long-term SiO eyes compared to PPV without long-term SiO and unoperated eyes (*p* = < 0.001).

**Conclusion:**

These findings suggest PPV with long-term SiO may alter the AS morphology of patients, its influence on the development of OAG remains unclear and warrants further investigation.

## Introduction

Glaucoma describes a condition in which there is progressive retinal ganglion cell loss leading to optic disc cupping and subsequent visual field defects, with 60–80 million individuals estimated to be affected by the condition [1, 2]. It is thought to occur due to poor drainage of the eye’s aqueous humour through outflow pathways, causing an increase in eye pressure.

Pars plana vitrectomy (PPV) is a surgical procedure involving the removal of vitreous humour in order to repair of numerous pathologies including: retinal detachment (RD), endophthalmitis, epiretinal membrane (ERM) and diabetic retinopathy (DR) [3]. PPV was first described in 1971 by Robert Machemer et al. [4] and has progressively become the procedure of choice for the management of retinal pathologies over the last two decades [5]. A study found the annual rates of vitrectomy in South Korea increased from 15.1 per 100,000 to 49.4 per 100,000 over an eleven-year period [6]. Similar trends have also been observed in Australia [7, 8]. From these findings it can be inferred we may see an increase in the prevalence of chronic glaucoma secondary to PPV in the future.

SiO is a term used to describe silicon-oxygen polymers and monomers that are hydrophobic in nature. This compounds specific gravity, buoyancy, interfacial tension and viscosity makes it an excellent tamponade agent in retinal surgery [9, 10]. Whilst SiO injection for tamponade in conjunction with PPV is considered a relatively safe and effective treatment modality, an increase in the development of glaucoma in the long-term has been reported [11].

In this characterization study we present the first study outlining angle morphology of a rare group of patients with long-term SiO in situ following PPV using Optical Coherence Tomography (OCT). We also aim to summarize the present understanding of the pathogenesis of late onset glaucoma secondary to SiO.

## Methods

### Study design

This study is a prospective, comparative characterization study. The study was approved by the Central Adelaide Local Health Network Human Research Ethics Committee** (**CALHN HREC) at the Royal Adelaide Hospital (RAH). Patients for inclusion in this study were identified through outpatient clinics at the RAH from January 2022 to July 2023. For this study the inclusion criteria required participants to be over the age of 18 and have undergone PPV. For eligibility to be included in the long-term SiO in situ group a patient must have had SiO 1300 centistokes in situ for at least 12 months at time of clinical assessment and image collection. Eyes eligible for inclusion in the without long-term SiO were eyes with SiO in situ for less than 3 months, air, gas or no tamponade. The exclusion criteria applied was: if patients developed post-operative complications (e.g. endophthalmitis) or if a subject’s eye was unable to be fully assessed (e.g. phthisical eye), have angles visualised on gonioscopy or anterior segment imaging was insufficient quality to collect all measurements.

After providing informed consent, patients underwent slit lamp examination by a single experienced consultant ophthalmologist. Data recorded included: any corneal findings, if angles were open, presence of SiO in angle quadrants, peripheral angle synechiae, macroscopic oil in the angles, cells in anterior chamber (AC), oil in the AC, oil on iris, peripheral iridotomy, emulsified oil in the posterior chamber (PC) and any significant fundus findings.

Following examination, patients underwent imaging of their anterior segment (AS) using the Zeiss Cirrus HD-OCT (Carl Zeiss, Dublin, California, USA) on the AS-OCT setting. Images of all patients were undertaken between 9am and 1pm to reduce the potential of any diurnal variation influencing collected results. All participants underwent imaging of their operated eye(s) and their fellow unoperated eye if possible. The collection of AS-OCT images was collected in the 3–9 O’clock meridians.

**Fig. 1:**
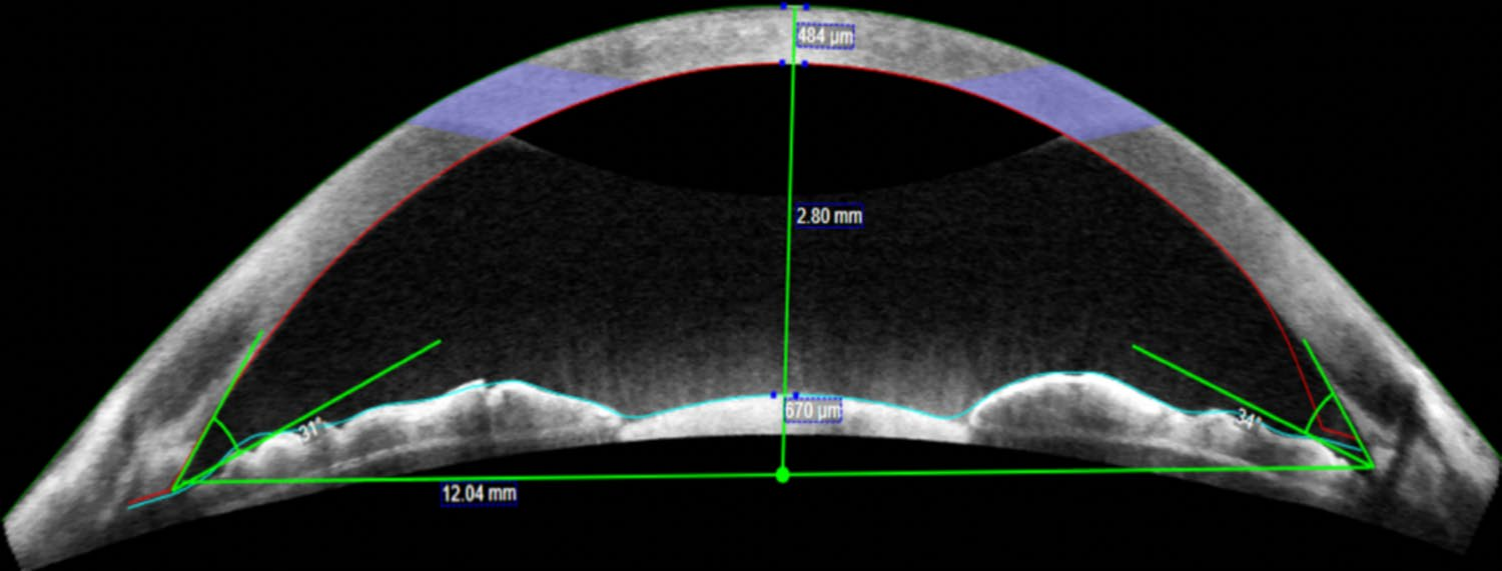
An AS-OCT image used for data collection

Software within the Zeiss Cirrus HD-OCT was used to measure: the central corneal thickness (CCT), anterior chamber diameter (ACd), anterior chamber depth (ACD), chamber area, temporal irideocorneal angle and nasal irideocorneal angle of every eye imaged (See Fig. 1). The CCT was generated automatically by the software, measuring the thickness in microns (µm) from the endothelium to the epithelium. The ACd was considered the distance between the two AC recesses in millimetres (mm). The ACD was deemed to be the distance measured from the corneal endothelium to the anterior lens capsule (if phakic) or intraocular lens optic surface (if pseudophakic) in mm. The chamber area was automatically calculated by the software in mm^2^, with no manual measurements required. The nasal and temporal iridocorneal angles were measured by measuring the angle between a point approximately 500 µm above the scleral spur to the corresponding point at the base of the iris. All measurements were repeated and confirmed by a second assessor during data collection.

A total of 42 patients were included in the study. There were 30 patients included who underwent PPV without long-term SiO in situ. From the 30 patients, three had unoperated eyes with poor segmentation of images which had to be excluded from the study and two patients had undergone PPV in both eyes. In the long-term SiO group a total of 12 patients were recruited for participation in this study. One patient had long-term silicone oil in both eyes and three patients were unable to have their unoperated eye images collected (one due to being unable to fixate of the target due to poor vision, two declined to have their unoperated eye imaged). There was a total of 13 eyes included within the operated eyes data and eight eyes were included in the unoperated data.

### Statistical analysis

Data was collated and summarized in Microsoft Excel. Statistical analysis was performed using SPSS (Statistical Pack for the Social Sciences) software (Version 29.0.1.0 (171), IBM Corporation, Armonk, NY, USA). Descriptive statistics are conveyed as means with standard deviations and categorical variables are conveyed as frequencies and percentages. The Shapiro–Wilk test was used to assess the normality of collected data for the overall dataset, the PPV only versus corresponding unoperated eye and long-term SiO in situ and corresponding unoperated eye group. Paired t-tests were used to compare normally distributed morphology variables in the PPV only versus unoperated eye and long-term SiO versus unoperated eye groups. For non-parametric variables, the Wilcoxon signed-rank test was used. A multivariate analysis of variance (MANOVA) was used to compare angle morphology measurements of unoperated eyes versus PPV without long-term SiO versus PPV with long-term SiO in situ. All assumptions for the MANOVA were met for all variables other than temporal angle in the pooled dataset analysis, therefore it was excluded.

## Results

In this study 78 eyes from 42 total patients were included, their mean age of 63.74 ± 19.13 years and 64.29% (27/42) of participants were males. In the long-term SiO in situ group the mean age of participants was 68.50 ± 20.03 years, with 50% of the participants being female. Comparatively, the mean age of the PPV without long-term SiO was 61.83 ± 18.77 years with 70% of participants being males.

The most common indication for PPV without long-term SiO were RD and vitreous haemorrhage (VH). In eyes with long-term SiO being left in situ was recurrent RD (see Table 1 for indications). In this study three eyes containing SiO developed glaucoma, requiring long-term intraocular pressure lowering therapy. In the PPV without SiO group, 62.5% of included eyes were pseudophakic at time of image collection. Comparatively, only 50% of long-term SiO in situ eyes were pseudophakic.

**Table 1:** Patient demographics and indications for PPV within recruited patients

Demographics	PPV without long-term SiO	PPV with long-term SiO
Mean age (years)	61.83 ± 18.77	68.50 ± 20.03
Males n(%)	21 (70)	6 (50)
Females n(%)	9 (30)	6 (50)
Phakic n(%)	12 (37.5)	7 (54)
Pseudophakic n(%)	20 (62.5)	6 (46)
*Tamponade agent*		
Air	2	
C3F8 (Octafluoropropane)	7	
SF6 (Sulphur hexafluoride)	7	
No tamponade	13	
SiO 1300	3	13
*Indications*		
Acute retinal necrosis and detachment		2
Giant retinal tear	1	–
Epiretinal membrane	2	–
Retinal Detachment	12	–
Recurrent Retinal detachment	–	7
Proliferative vitreoretinopathy related re-detachment	–	3
FTMH	3	–
Vitreous Haemorrhage	6	–
Subretinal Haemorrhage	1	–
Iris Chafing causing UGH	1	–
Retained lens matter	3	–
Vitreomacular traction	1	–
Subluxed IOL	2	–
Other	–	1
Total	32	13

For patients who underwent PPV without long-term SiO with a corresponding unoperated eye, there was no statistical difference in mean CCT, ACd, ACD, chamber area, nasal or temporal or iridocorneal angle (See Table 2). In the operated PPV without long-term SiO group, the mean CCT (556.48 ± 36.52 vs. 556.92 ± 45.1 µm) and nasal iridocorneal angle (34.24 ± 7.74 vs. 34.76 ± 8.68 degrees) measurements were smaller means than corresponding unoperated eyes.

**Table 2:** Mean morphology measurements of PPV without long-term SiO versus fellow unoperated eyes

Variable	Mean difference	Std. deviation difference	Mean (operated)	Std. deviation (operated)	Mean (unoperated)	Std. deviation (unoperated)	Std. error mean difference	95% CI lower bound difference	95% CI upper bound difference	t-value	df	One-sided *p* value	Two-sided *p* value
CCT	− 0.44	30.13	556.48	36.52	556.92	45.10	6.03	− 12.88	12	− 0.07	24	0.47	0.94
ACd	0.04	0.53	12.53	0.73	12.49	0.59	0.11	− 0.18	0.26	0.4	24	0.35	0.69
ACD	0.09	0.48	3.11	0.49	3.02	0.60	0.1	− 0.11	0.29	0.97	24	0.17	0.34
Chamber area	1.21	4.73	25.35	4.22	24.14	5.29	0.95	− 0.74	3.16	1.28	24	0.11	0.21
Nasal angle	− 0.52	8.55	34.24	7.74	34.76	8.68	1.71	− 4.05	3.01	− 0.3	24	0.38	0.76
Temporal angle	1	1.76	31.84	10.31	30.84	8.42	–	–	–	–	–	–	0.77*

When evaluating patients with PPV with long-term SiO in situ and a corresponding unoperated control eye, the same trend was observed with no statistical difference in AS morphology measurements between the two groups (see Table 3). A comparison of these groups illustrated operated eyes had a larger mean CCT by 7.75 μm, ACD by 0.32 mm, temporal irideocorneal angle by 7.38 degrees and nasal irideocorneal angle by 6.25 degrees compared to control eyes.

**Table 3:** Mean morphology measurements of PPV with long-term SiO versus fellow unoperated eyes

Variable	Mean difference	Std. deviation difference	Mean (oil operated)	Std. deviation (oil operated)	Mean (unoperated)	Std. deviation (unoperated)	Std. Error mean difference	95% CI lower bound difference	95% CI upper bound difference	t-value	df	One-sided *p* value	Two-sided *p* value
CCT	7.75	53.78	544.63	39.05	536.88	44.54	19.01	− 37.21	52.71	0.41	7	0.35	0.7
ACd	− 0.26	0.51	11.84	0.43	12.1	0.59	0.18	− 0.69	0.17	− 1.45	7	0.10	0.19
ACD	0.32	0.87	3.24	0.39	2.93	0.77	0.31	− 0.41	1.05	1.03	7	0.17	0.34
Temporal angle	7.38	14.76	38.5	8.91	31.13	8.59	5.22	− 4.97	19.72	1.41	7	0.10	0.20
Nasal angle	6.25	14.38	40.38	8.19	34.13	12.25	5.08	− 5.77	18.27	1.23	7	0.13	0.26
Chamber area	− 2.05	4.59	24.91	2.92	22.85	8.64	–	–	–	–	–	–	0.58*

The MANOVA of all pooled data found the ACd to be statistically significant between the three groups (*p* = < 0.001). In the analysis, patients who underwent PPV with long-term SiO in situ had the smallest mean ACd, followed by PPV without long-term SiO and unoperated eyes, respectively (See Table 4).

**Table 4:** Mean morphology measurements of pooled data comparing PPV with long-term SiO versus PPV versus unoperated eyes

Variable	Group	Mean	Standard deviation	95% CI lower bound	95% CI upper bound	N	*p* value
CCT	PPV with Long-term SiO	568.15	55.65			13	0.534
	PPV without Long-term SiO	549.94	53.99			32	
	Unoperated eyes	552.06	45.12			33	
	Total	553.87	50.44	542.50	565.25	78	
ACd	PPV with Long-term SiO	11.75	0.41			13	< .001
	PPV without Long-term SiO	12.53	0.68			32	
	Unoperated eyes	12.39	0.60			33	
	Total	12.34	0.66	12.19	12.49	78	
ACD	PPV with Long-term SiO	3.12	0.44			13	0.384
	PPV without Long-term SiO	3.19	0.52			32	
	Unoperated eyes	2.99	0.63			33	
	Total	3.1	0.56	2.97	3.22	78	
Chamber Area	PPV with Long-term SiO	23.54	4.52			13	0.14
	PPV without Long-term SiO	26.22	4.75			32	
	Unoperated eyes	23.83	6.13			33	
	Total	24.76	5.42	23.54	25.98	78	
Nasal Angle	PPV with Long-term SiO	40.69	8.49			13	0.10
	PPV without Long-term SiO	35.53	7.89			32	
	Unoperated eyes	34.61	9.46			33	
	Total	36	8.84	34.01	37.99	78	

Clinical examination of the 13 operated eyes with long-term SiO demonstrated five eyes contained emulsified SiO within angles (see Table 5 for quadrant distribution of oil). Eleven eyes were noted to have quadrant angles open on gonioscopy and three eyes had macroscopic oil in the AC.

**Table 5:** Number/proportion of operated eyes with emulsified oil on clinical examination (n = 13)

	Emulsified oil in AC	Oil in superior quadrant	Oil in inferior quadrant	Oil in nasal quadrant	Oil in temporal quadrant	Open quadrant angles	Macroscopic oil	Cells in AC	Oil in AC	Oil in Iris
Number of eyes	5	6	1	5	5	8	3	0	2	4
Percentage of eyes (%)	38.46	46.15	7.69	38.46	38.46	61.54	23.08	0.00	15.38	30.77

## Discussion

The current contribution of SiO endotamponade following PPV to the development of glaucoma long-term is limited. The findings in this study suggest long-term silicone oil situ may alter the morphology of the AC.

Prior to the use of OCT for anterior segment imaging, ultrasound biomicroscopy (UBM) was often the imaging modality of choice for collecting high-definition images of the anterior segment [12]. UBM allows for the generation of quality images of intraocular structures through the use of 50–100 MHz high frequency soundwaves [13]. In 2006, Margio et al. [14] compared the AC morphometric parameters of eyes undergoing PPV pre-operatively and eight weeks post-operatively, finding no significant differences in parameters including ACD and angle opening distance. Comparatively, another study comparing pre-operative parameters to the immediate post-operative period noted ACD was only reduced in patients having undergone gas tamponade and there was no correlation between ACD measurements and IOP elevation [15].

Li et al. [16] compared ACD’s of PPV patients using A-scan and found no mean ACD difference in eyes that underwent PPV for VH in the three month post-operative period. Interestingly, they noted eyes with ERMs had a deeper ACD pre-operatively compared to their fellow eye, this phenomenon was observed to persist one week post procedure but resolved at subsequent follow up. This suggested ERM induced ACD deepening was reversible with successful vitrectomy.

Another study found no difference in AC volume and iridocorneal angle following either PPV alone or PPV with SiO injection [17]. At the one week follow up they did however note an increase in the ACD of the SiO injected group and a decrease in the ACD of the PPV without SiO group. Furthermore, the SiO group had a reduction in CCTs during the one month post-operative follow up period. Ünsal et al.’s [18] UBM evaluation of found PPV patients with SiO had reductions in trabecular meshwork-ciliary process distance and iris-ciliary process distance. Similar results were also observed for their gas tamponade cohort, with an additional statistically significant reduction in ACD. In this study the authors concluded gas tamponade patients had a greater alteration to AC morphology, suggesting this attributes to the greater incidence of IOP rise in this specific sup-group.

Ghomi et al. [19] found although no significant change in AC parameters following undergoing vitrectomy, there was an increase in anterior–posterior lens size. They attributed this to an increase in lens opacity secondary to PPV. Similarly, a study of phakic patients with mac-off retinal detachments had an increase in axial length, ACD and lens thickness [20]. A more recent study reported patients undergoing PPV with indented vitreous base shaving were noted to have an increase in ACD and irideocorneal angle over a 3 month post-operative period [21].

Literature using AS-OCT for the assessment of anterior morphology is far more limited. One study analysed 245 eyes prospectively noting a reduction in both ACD in the 12-month post-operative period for both patients undergoing PPV and SB. Further to this, SB patients were noted to also have a mean increase in axial length [22]. In contrast, Khodabande et al. [23] found no difference in ACD, ACd, irideocorneal angle size in patients who underwent PPV without tamponade. Worth noting, axial length changes have been noted post vitrectomy in patients with pre-operative hypotony or extreme myopia, however it remains unclear whether this translates to changes within the anterior segment [24].

The influence of PPV and long-term SiO is not entirely established, with variable morphological changes reported in literature thus far. Therefore, the precise aetiologic mechanism by which it contributes to the long-term development of glaucoma remains a topical issue. In 1988, Burk et al. [25] suggested intravitreal SiO may play a role in the development of late onset glaucoma through its presence in the AC angles. They undertook histopathology testing of six enucleated eyes, noting the presence of: fibrovascular membranes, inflammatory cells or cellular debris in both AC angles and the trabecular meshwork. Gao et al. [26] also suggested a link between SiO in the AC and IOP elevation.

Following this, one study analysed the angles of patients with SiO in situ using gonioscopy, noting at least 50% of eyes had emulsified oil present in angles regardless of SiO density injected [27]. This study found oil was predominately noted in the 1–4 O’clock areas of the superior trabecular meshwork. Subsequent to this, an evaluation of trabecular meshworks via electron microscopy detected fibrosis/thickening of trabecular spaces and narrowing of intertrabecular spaces in SiO patients [28]. Microscopic analysis also demonstrated the presence of cellular debris within the meshwork, however there were no SiO bubbles or macrophages suggestive of inflammation noted. In contrast, ultrasound biomicroscopy has demonstrated the presence of silicone oil bubbles in patients’ trabecular meshworks, peripheral synechiae were also noted [29].

In context of the above, one of the main hypotheses at present suggests SiO is thought to aggregate at the trabecular meshwork and form a physical barrier for aqueous outflow [30]. This is thought to occur over time as a result of SiO bubbles (being injected for tamponade following vitrectomy) migrating into the AC. Much more recently, a case report identified the presence of SiO in Schlemm’s canal on AS-OCT, again suggesting the possibility of SiO being involved with the development of glaucoma [31].

There is also a possibility the presence of SiO in the AC may elicit an inflammatory response, which in turn alters the structure and impedes the function of aqueous drainage pathways. Liu et al. [32] study comparing patients with and without glaucoma following SiO injection demonstrated glaucoma group had significantly higher levels levels of IL-17, IL-6 and TNF-α in their aqueous humour. Surprisingly, although the glaucoma group had a greater proportion of patients with SiO in the AC, slit lamp examination demonstrated the control group had a greater proportion of patients with emulsion overall (both results were not statistically significant). Semeraro et al. [33] compared the inflammatory activity of patients receiving standard SiO to heavy oil, they reported higher prostaglandin E2 and interleukin-1α in the heavy SiO group.

### Limitations

A limitation of this study is the sample size of long-term SiO patients, there were only 13 operated and 8 control eyes within this study. This is due to long-term silicone oil rarely being left in situ for PPV patients and thus a very limited proportion of clinic patients being eligible to participate in the study. In future, to validate the results of this study, a larger multicentre trial is likely required. Furthermore, future studies would benefit from ensuring all participants having pre-operative AS-OCT imaging to allow for a pre-operative comparison, although it is often difficult to predict which patients may require long-term oil indefinitely.

Another limitation of this study is the image collecting limitations of the AS-OCT. This imaging modality can be operator dependent and often given subjects had low vision-there were challenges with fixation on specified targets whilst collecting images. Furthermore, whilst the software within the Zeiss HD-OCT software is accurate, it still requires human intervention to set parameters for some measurements, thereby introducing a possibility of human error. To mitigate this, all measurements were undertaken by a single investigator.

## Conclusion

The present mechanisms relating to the development of late onset glaucoma following PPV and long term SiO in situ are SiO forming a physical barrier to drainage and SiO triggering an inflammatory response in the anterior segment. This characterization study is to our knowledge, the first study reporting the angle morphology of PPV patients with long-term SiO in situ longer than 12 months using AS-OCT. Furthermore, AS-OCT appears to be a valuable clinical tool to assist ophthalmologists during angle assessment. Data from this rare patient group suggests SiO alters the size of the anterior segment, this may in turn interfere with the production or drainage of aqueous humour and contribute to the development of late onset glaucoma.

## Data Availability

Data is summarised within the manuscript. Please contact the corresponding author directly for patient data relating to the study findings.
